# From Screens to Sheets: Understanding Social Media Addiction, Online Sexual Activity, Pornography Consumption, and Sexual Satisfaction Among Greek Adults

**DOI:** 10.7759/cureus.98947

**Published:** 2025-12-11

**Authors:** Iliana Braouzou, Krystallia Gkouletsa

**Affiliations:** 1 Department of Communication and Media Studies, National and Kapodistrian University of Athens, Athens, GRC; 2 Department of Nursing, University of Thessaly, Larissa, GRC; 3 Department of Urology, University of Thessaly, Larissa, GRC

**Keywords:** greek adults, online sexual activity, pornography, sexual satisfaction, social media addiction

## Abstract

Introduction: Digital technologies increasingly shape contemporary sexual behaviors, with social media use, online sexual activity, and pornography consumption becoming common aspects of modern intimacy.

Aim: This study examined the relationships between social media addiction, online sexual activity, problematic pornography consumption, and sexual satisfaction among Greek adults and explored how these associations vary across demographic groups.

Materials and methods: A quantitative, cross-sectional correlational design was employed with a sample of 676 Greek adults recruited via online platforms. Participants completed an anonymous questionnaire including the Bergen Social Media Addiction Scale, Internet Sex Screening Test, Problematic Pornography Consumption Scale, and Satisfaction With Sex Life Scale. Nonparametric tests (Spearman rank correlation, Kruskal-Wallis tests) and simple linear regressions were conducted using SPSS Statistics version 25 (IBM Corp. Released 2017. IBM SPSS Statistics for Windows, Version 25.0. Armonk, NY: IBM Corp.).

Results: Higher levels of social media addiction, online sexual activity, and problematic pornography consumption were each significantly associated with lower sexual satisfaction (rs = -0.230, rs = -0.148, and rs = -0.293, respectively; all p < 0.001). Social media addiction positively predicted online sexual activity (β = 0.356, p < 0.001; R² = 0.127) and problematic pornography consumption (β = 0.407, p < 0.001; R² = 0.165), while negatively predicting sexual satisfaction (β = -0.244, p < 0.001; R² = 0.060). Online sexual activity strongly predicted problematic pornography consumption (β = 0.748, p < 0.001; R² = 0.559) and also predicted lower sexual satisfaction (β = -0.215, p < 0.001; R² = 0.046). Demographic analyses showed that transgender participants, younger individuals, and those with lower educational attainment reported higher levels of social media addiction (p < 0.05), online sexual activity (p < 0.05), and problematic pornography use (p < 0.05). Regarding sexual orientation, bisexual and pansexual participants demonstrated the lowest sexual satisfaction, whereas homosexual participants reported the highest levels of sexual satisfaction (p < 0.001).

Conclusions: The findings suggest that excessive engagement in digital sexual behaviors and problematic online media use are linked to reduced sexual satisfaction in Greek adults. These associations appear consistent across demographic groups but are more pronounced in specific populations. The results underscore the need for culturally tailored interventions promoting balanced digital engagement, sexual health education, and awareness of the potential psychological and relational impacts of online sexual behaviors.

## Introduction

The digital revolution has transformed human interaction, significantly influencing how individuals establish, maintain, and express intimacy [1]. Among the most pervasive outcomes of this transformation is the widespread adoption of social media platforms and online sexual content, including pornography, as primary venues for sexual exploration and gratification [2]. While digital technologies can enrich relational experiences, growing concerns surround their potential to displace offline intimacy and reshape sexual behaviors and expectations [3,4]. These developments highlight the need to distinguish between different forms of digital engagement, such as social media use, online sexual interactions, and pornography consumption, as each may exert unique yet interconnected influences on intimacy and sexual well-being.

Social media addiction, characterized by excessive concern with and use of social platforms to the detriment of other life domains, is increasingly recognized as a behavioral addiction [5]. Though initially studied in the context of mental health, emerging research now investigates its implications for intimate relationships and sexual well-being [6]. Simultaneously, pornography consumption has become both more accessible and normalized through digital means, raising questions about its effects on sexual satisfaction and expectations [7,8]. Conceptually, both behaviors share standard psychological mechanisms, such as reward-seeking, emotional regulation, and novelty pursuit, which may help explain why they often co-occur and how they collectively influence sexual satisfaction [9].

Moreover, online sexual activity, ranging from sexting and video sex to engaging in cybersex with strangers, has emerged as a distinct behavioral pattern, often intersecting with both social media use and pornography consumption [10]. Online sexual activity can function as a behavioral bridge between the social and erotic domains of digital life, making it an essential construct for understanding how individuals navigate intimacy in technologically mediated environments [10]. Despite growing empirical interest in these behaviors [11], most research has focused on adolescents and Anglo-American contexts, often overlooking cultural dimensions that may mediate or moderate these phenomena [12,13]. This Western-centric focus risks universalizing findings that may be culturally specific, neglecting how sociocultural norms, religious values, and gender scripts shape both online sexual behaviors and their interpretations. In Greece, where traditional and modern values coexist in tension, the dynamics between digital sexual behaviors and personal satisfaction warrant culturally specific investigation [14]. Greek society is shaped by strong family structures, Orthodox Christian influences, and evolving attitudes toward sexuality, creating a unique cultural backdrop in which digital sexual behaviors may carry different meanings, motivations, and psychosocial consequences compared to other Western contexts. Understanding these cultural nuances is essential for interpreting digital intimacy patterns and for explaining why specific populations within Greece may be more or less vulnerable to problematic online sexual behavior.

Social media addiction and its impact on sexual intimacy

Social media has become a central component of daily life, altering the ways individuals communicate, present themselves, and engage in relationships [1]. While social networking platforms provide valuable opportunities for interpersonal connection, a growing body of research has examined the darker side of this integration, namely, problematic or addictive use [5]. Social media addiction has been defined as excessive preoccupation with or use of social networking services that leads to impairments in functioning, withdrawal symptoms, and loss of control [6]. This phenomenon has been associated with reduced psychological well-being, increased loneliness, and difficulty in maintaining offline relationships [15,16]. Recent studies indicate that social media addiction affects approximately 5-18% of users globally, with higher rates reported among young adults [17-19].

In the context of romantic and sexual relationships, problematic social media use may be particularly disruptive. Studies [20,21] have found that high levels of engagement with platforms such as Instagram and Facebook correlate with lower levels of relationship satisfaction, often due to jealousy, surveillance behaviors, and emotional distance. Time spent online can displace time spent engaging in physical intimacy or emotionally bonding with a partner [22]. Furthermore, persistent exposure to idealized images of other people’s bodies and relationships can lead to negative self-perceptions and dissatisfaction with one’s own sex life or partner, particularly among women [23,24].

In terms of individual sexual well-being, some research suggests that compulsive engagement with social media may negatively affect sexual desire and self-esteem, especially when it triggers social comparison or body image concerns [25]. Users may also turn to social media as a form of validation-seeking or escape from offline sexual dissatisfaction, which may reinforce a cycle of avoidance and disconnection from real-life intimacy [26]. These patterns appear more pronounced in individuals with underlying anxiety, attachment insecurity, or low sexual assertiveness [27].

Online sexual activity and digitalization of sexual experience

The digitization of sexuality has introduced new forms of erotic expression, including sexting, video sex, erotic chat, and interactive sexual experiences through webcams or virtual platforms. These practices are increasingly considered normative, especially among adolescents and young adults who view digital sexual expression as an extension of contemporary intimacy [10,28]. The frequency and diversity of these behaviors have grown with mobile technologies and app-based interactions, such as those facilitated by dating apps and encrypted messaging platforms [29].

Cross-cultural research has begun to show that patterns of online sexual behavior differ meaningfully between countries. For example, a study [30] comparing American and Spanish university students found that American students engaged in sexting more frequently and were more likely to experience non-consensual dissemination of sexual images. In contrast, Spanish students reported receiving more sexts overall. Similarly, a cross-national comparison of U.S. and Peruvian college students showed that Peruvian students from a more traditional, Catholic context reported higher levels of online sexual activity, possibly using digital environments to navigate stricter offline norms around sexuality [31]. More recent work [32] has reported distinct sexting frequencies and motives among emerging adults from different Hispanic countries, further underscoring the role of sociocultural values in shaping digital sexual behavior. These cross-cultural findings highlight that online sexual behaviors are shaped not only by technological affordances but also by local norms, gender expectations, and cultural attitudes toward sexuality, which influence how individuals interpret and engage in digital sexual interactions. However, most existing studies examine these behaviors separately or within culturally narrow samples, limiting the ability to draw broader conclusions about how online sexual activity operates across genders, relationship contexts, and sociocultural environments.

Online sexual engagement has been linked to various psychological and relational outcomes, depending on context, frequency, and individual motivations. Some studies [33,34] suggest that when practiced consensually and within emotionally secure relationships, online sexual communication can strengthen intimacy, increase sexual satisfaction, and promote sexual openness between partners. It has also been shown to support long-distance couples in maintaining sexual connection and reduce sexual boredom by allowing exploration of fantasies or preferences that may not be expressed offline [35].

Conversely, there is evidence that compulsive or secretive participation in online sexual interactions, particularly with strangers or outside of committed relationships, may correlate with diminished sexual satisfaction and relational strain [8,36,37]. For some individuals, online sexual behavior may serve as a coping strategy for dealing with dissatisfaction in their offline sex life or interpersonal insecurities, rather than functioning as a complement to healthy sexuality [38]. In such cases, these activities may foster avoidance of real-life sexual encounters, reinforce unrealistic expectations, and lead to guilt or emotional detachment [39].

Finally, the role of gender is vital in shaping how online sexual experiences are perceived and internalized. Research [2,40] has shown that men and women differ in their frequency of engagement, motivations, and emotional responses to digital sexual interactions. Women are more likely to engage in such behavior within the context of relationships or emotional connection, while men may seek novelty, excitement, or anonymous gratification.

Pornography use and its effects on sexual satisfaction

Pornography consumption has become a highly prevalent sexual behavior, made increasingly accessible by the internet’s affordability, anonymity, and convenience [41]. Recent studies estimate that 70-95% of men and 50-80% of women report having consumed pornography, with particularly high rates observed among adolescents and young adults [41-44]. Additionally, more than 25% of adults report viewing pornography at least once per week, indicating not only widespread lifetime exposure but also frequent ongoing use [43,44]. While some scholars [7] regard pornography as a legitimate outlet for sexual exploration and arousal, others [45] have raised concerns about its effects on sexual expectations, satisfaction, and relational dynamics. Research [7,45] has demonstrated a complex and often contradictory relationship between pornography use and sexual satisfaction, moderated by variables such as gender, relationship status, frequency of use, and reasons for consumption. Theoretical models such as sexual script theory [46] and compensatory Internet use theory [47] help explain why digital sexual behaviors may emerge: individuals may turn to online environments to fulfill expectations shaped by media, or to cope with unmet emotional or relational needs offline.

In numerous studies [8,36,48], frequent solo consumption of pornography, particularly in the absence of shared sexual activity with a partner, has been associated with lower sexual satisfaction and increased dissatisfaction with one’s partner’s sexual performance or physical appearance. The internalization of unrealistic sexual scenarios, objectified bodies, and instant gratification models may lead to misaligned sexual expectations and reduced arousal during real-life intimacy [49]. Moreover, repeated use may contribute to desensitization, a decrease in novelty, and performance anxiety, especially among men [45]. These positive outcomes illustrate that digital sexual interactions can function as adaptive extensions of intimacy when embedded within stable relational dynamics.

However, other scholars [50] have highlighted the potential for pornography to enhance sexual experiences, particularly when consumed by couples or used to initiate communication about desires, fantasies, or preferences. Some individuals report increased sexual satisfaction and greater comfort with their own sexuality after incorporating pornography into their sexual routines. In these cases, the relational context, mutual consent, and alignment in sexual values between partners are key in determining whether pornography use enhances or hinders satisfaction [37]. Such findings suggest that the impact of online sexual activity is highly context-dependent and that its maladaptive manifestations may be best understood through frameworks addressing problematic or addictive online behaviors.

Cultural and religious norms also mediate the emotional and moral reactions to pornography use. In societies where sexuality remains closely tied to moral or religious expectations, pornography use may provoke feelings of guilt, secrecy, or conflict with internalized beliefs [51]. These reactions may exacerbate the adverse effects on sexual well-being, especially among individuals who lack access to alternative models of sexual discourse [51]. Gender differences also persist; women tend to report more ambivalence or discomfort with pornography, particularly when its themes reinforce misogynistic or violent content [52].

Aim

This study aims to investigate the relationships among social media addiction, online sexual activity, pornography consumption, and sexual satisfaction among Greek adults. By examining these interconnected digital behaviors within a specific cultural context, the study seeks to clarify how contemporary online practices influence individuals’ sexual well-being and to contribute empirical evidence from Greece to address gaps in the existing literature on digital intimacy.

## Materials and methods

Research design and data collection

This study employed a quantitative, cross-sectional correlational design to investigate the relationships among social media addiction, online sexual activity, problematic pornography consumption, and sexual satisfaction among adults in Greece. Data were collected through an anonymous, self-administered online questionnaire. The questionnaire had five sections, and some psychometric scales were used. The first section concerned demographic characteristics (gender, age, educational level, and sexual orientation) and consisted of four closed-ended questions.

The second section concerned social media addiction, and Bergen's Social Media Addiction Scale was used. The scale was created by Andreassen et al. [53] and consists of six items rated on a Likert scale from 1 (very rarely) to 5 (very often). A final score is calculated as the sum of responses, with a minimum possible score of 6 and a maximum possible score of 30; higher scores indicate greater addiction. The scale in the present study had high reliability (α = 0.839).

The third section concerned online sexual activity, and for this reason, the Internet Sex Screening Test was used. The scale was created by Delmonico and Miller [54] and consists of 25 items that participants evaluate as correct (yes) or incorrect (no). The scale examines the following dimensions: online sexual compulsivity, online sexual behavior-social, online sexual behavior-isolated, online sexual spending, and interest in online sexual behavior. Online sexual compulsivity consists of seven items (score range 0-7), online sexual behavior-social consists of six items (0-6), online sexual behavior-isolated consists of six items (0-6), online sexual spending consists of three items (0-3), and interest in online sexual behavior consists of three items (0-3). The total score is obtained by summing all 25 items (overall range 0-25), with higher scores indicating more problematic online sexual activity. The scale in the present study had very high reliability (α = 0.895). All scale dimensions also had high reliability, with alpha coefficients ranging from 0.708 (interest in online sexual behavior) to 0.886 (online sexual behavior-isolated).

The fourth section concerned pornography use, and the Problematic Pornography Consumption Scale was used. The scale was created by Bőthe et al. [55] and consists of 18 items rated on a Likert scale from 1 (never) to 5 (all the time). The scale examines six dimensions: salience, mood modification, conflict, tolerance, relapse, and withdrawal, each comprising three items. Thus, each dimension has a possible score range of 3 to 15, and the total PPCS score ranges from 18 to 90, with higher scores indicating more problematic pornography use. The scale in this study had very high reliability (α = 0.976). All dimensions of the scale also had high reliability, with alpha coefficients ranging from 0.864 (conflict) to 0.945 (withdrawal).

Finally, the fifth section concerned satisfaction with sexual life, and the Satisfaction With Sex Life Scale was used. The scale was created by Neto [56] and consists of five items that are rated on a Likert scale from 1 (strongly disagree) to 7 (strongly agree). A final score is obtained by summing the responses, yielding a minimum possible score of 5 and a maximum possible score of 35, with higher scores indicating greater satisfaction with the sex life. The scale in the present study had very high reliability (α = 0.956).

All four instruments used in this study, the Bergen Social Media Addiction Scale, the Internet Sex Screening Test, the Problematic Pornography Consumption Scale, and the Satisfaction With Sex Life Scale, are copyright-protected. Formal permission to use these scales was obtained from the respective copyright holders prior to data collection.

Sample

An a priori power analysis was conducted using G*Power 3.1.9.7 (Heinrich‑Heine‑Universität Düsseldorf, Düsseldorf, Germany) to determine the required sample size for the primary regression analysis. Assuming a medium effect size (f² = 0.15), an α level of 0.05, and power (1-β) = 0.80 with one predictor, the analysis indicated that a minimum sample of approximately 55 participants was required.

The inclusion criteria were (a) being 18 years of age or older and (b) being able to read and understand Greek to complete the questionnaire. Exclusion criteria included (a) being under 18 years old, (b) incomplete survey responses, and (c) inability to understand Greek sufficiently to answer the questionnaire.

The final sample consisted of 676 Greek adults, substantially exceeding the required minimum and thereby ensuring robust statistical power for all analyses; a convenience sampling approach was used.

Procedure

Data for this study were collected through a self-administered online questionnaire created using Google Forms (Google LLC, Mountain View, CA, USA). The questionnaire was distributed over nine months, from September 2024 to May 2025, allowing adequate time for participant recruitment and broad demographic reach.

The researcher personally distributed the survey link through personal social media accounts (Facebook and Instagram). This non-probability, convenience sampling approach aimed to attract a diverse adult population residing in Greece. The online format was selected for its accessibility, ease of use, and ability to preserve participant anonymity, critical given the sensitive nature of the topics explored. It is worth noting that the questionnaire was created and completed in Greek.

In accordance with international ethical standards for minimal-risk research, the study met the criteria for exemption from formal institutional review board (IRB) review. However, on the first page of the questionnaire, participants were presented with a research information and consent statement outlining the study's purpose, procedures, and ethical safeguards. Moreover, participants were informed that the results would be used exclusively for research purposes, including publications in journals. Participants were assured that their responses were completely anonymous and strictly confidential. That participation was entirely voluntary, with the option to withdraw from the survey at any point without consequence. No personally identifying information was collected, and responses were recorded in a format that precluded any form of participant identification. Data were automatically coded through Google Forms and securely stored in password-protected files accessible only to the researcher. In compliance with the General Data Protection Regulation, participants were informed that their data would be retained for four years and then permanently deleted. The questionnaire was designed to take approximately 10 minutes to complete, minimizing participant burden. A contact email was provided for participants to ask questions or request further information about the study. This procedure ensured informed consent, data protection, and minimal risk to participants, in line with ethical research practices in the social sciences.

Statistical analysis

SPSS Statistics version 25 (IBM Corp. Released 2017. IBM SPSS Statistics for Windows, Version 25.0. Armonk, NY: IBM Corp.) was used for statistical analysis of the data. Descriptive statistics were used to present the demographic characteristics of the participants as well as the levels of social media addiction, problematic online sexual activity, problematic pornography consumption, and satisfaction with sexual life. The reliability of the psychometric tools was checked using Cronbach’s alpha. After the distributional tests using Kolmogorov-Smirnov and Shapiro-Wilk, the data did not follow a normal distribution (p < 0.05); therefore, nonparametric tests were used. Specifically, Spearman correlations were used to test associations between social media addiction, problematic online sexual activity, problematic pornography consumption, and satisfaction with sexual life. Kruskal-Wallis tests were also used to investigate differences in levels of social media addiction, problematic online sexual activity, problematic pornography consumption, and sexual life satisfaction by demographic characteristics. Finally, simple linear regressions were conducted to examine the effects of social media addiction, problematic online sexual activity, problematic pornography consumption, and sexual life satisfaction. Statistical significance was defined as p < 0.05.

## Results

Demographic characteristics

In the total sample of 676 participants, 37.9% (n = 256) were male, 59.8% (n = 404) were female, and 2.4% (n = 16) were transgender. Also, 29% of the sample (n = 196) were 18-25 years old, 13.6% (n = 92) were 26-30 years old, 16% (n = 108) were 31-35 years old, another 16% of the sample (n = 108) were 36-40 years old, while 3.6% (n = 24) were 41-45 years old, 8.9% (n = 60) were 46-50 years old, and 13% (n = 88) were over 50 years old. Additionally, 34.9% (n = 236) were high school graduates, 40.2% (n = 272) were university graduates, and 24.9% (n = 168) had a master's degree. Finally, 85.8% (n = 580) were heterosexual, while 3.6% (n = 24) were homosexual, 5.9% (n = 40) were bisexual, and 4.7% (n = 32) were pansexual (Table 1).

**Table 1:** Demographic characteristics N: 676, n: frequency, %: percentage

Demographic characteristic	n	%
Gender		
Male	256	37.9
Female	404	59.8
Transsexual	16	2.4
Age		
18-25	196	29.0
26-30	92	13.6
31-35	108	16.0
36-40	108	16.0
41-45	24	3.6
46-50	60	8.9
50+	88	13.0
Educational level		
High school graduate	236	34.9
University graduate	272	40.2
Master's degree holder	168	24.9
Sexual orientation		
Heterosexual	580	85.8
Homosexual	24	3.6
Bisexual	40	5.9
Pansexual	32	4.7

Levels of social media addiction, online sexual activity, problematic pornography use, and satisfaction with sex life

Participants had moderate levels of social media addiction (M = 14.75, SD = 5.36) and low-to-moderate levels of online sexual activity (M = 6.69, SD = 5.37). Subscale scores showed low interest in online sexual behavior (M = 0.67, SD = 1.00), low online sexual spending (M = 0.74, SD = 0.90), moderate engagement in online sexual behavior-isolated (M = 2.83, SD = 1.95), low-to-moderate online sexual behavior-social (M = 1.53, SD = 1.56), and low online sexual compulsivity (M = 0.91, SD = 1.31). Participants also reported moderate levels of problematic pornography consumption (M = 34.66, SD = 23.77). Subscale scores indicated low-to-moderate salience (M = 6.23, SD = 4.51), moderate mood modification (M = 7.38, SD = 5.01), low conflict (M = 5.19, SD = 3.72), low tolerance (M = 5.73, SD = 4.48), low relapse (M = 5.20, SD = 4.04), and low withdrawal (M = 4.93, SD = 3.92). Finally, participants reported moderate levels of satisfaction with their sex life (M = 20.81, SD = 9.29) (Table 2).

**Table 2:** Means and standard deviations for social media addiction, online sexual activity, problematic pornography use, and satisfaction with sex life N: 676, M: mean, SD: standard deviation

Variable	M	SD
Social media addiction	14.75	5.36
Online sexual activity	6.69	5.37
Interest in online sexual behavior	0.67	1.00
Online sexual spending	0.74	0.90
Online sexual behavior-isolated	2.83	1.95
Online sexual behavior-social	1.53	1.56
Online sexual compulsivity	0.91	1.31
Problematic pornography consumption	34.66	23.77
Salience	6.23	4.51
Mood modification	7.38	5.01
Conflict	5.19	3.72
Tolerance	5.73	4.48
Relapse	5.20	4.04
Withdrawal	4.93	3.92
Satisfaction with sex life	20.81	9.29

Differences in social media addiction, online sexual activity, problematic pornography use, and satisfaction with sex life according to demographic characteristics

Regarding gender, transsexual participants reported the highest levels of social media addiction (M = 20.75, SD = 4.92), followed by females (M = 14.89, SD = 5.15) and males (M = 14.16, SD = 5.50) (p < 0.001). Online sexual activity was also highest among transsexuals (M = 16.75, SD = 7.53), followed by males (M = 9.78, SD = 5.34) and females (M = 4.33, SD = 3.55) (p < 0.001). Similarly, transsexual participants exhibited the highest levels of problematic pornography consumption (M = 75.25, SD = 37.00), followed by males (M = 46.95, SD = 28.54) and females (M = 25.27, SD = 11.42) (p < 0.001). A notable divergence was observed in satisfaction with sex life: females reported the highest satisfaction (M = 22.23, SD = 9.38), followed by males (M = 19.28, SD = 8.71), whereas transsexual participants reported the lowest levels of satisfaction (M = 9.50, SD = 2.37) (p < 0.001) (Table 3).

**Table 3:** Gender differences in social media addiction, online sexual activity, problematic pornography use, and satisfaction with sex life N: 676, n: frequency, M: mean, SD: standard deviation, H: H-statistic (df = 2), *: Kruskal-Wallis

	Gender	n	M	SD	H (2)	p*
Social media addiction	Male	256	14.16	5.50	22.179	<0.001
	Female	404	14.89	5.15		
	Transsexual	16	20.75	4.92		
Online sexual activity	Male	256	9.78	5.34	194.285	<0.001
	Female	404	4.33	3.55		
	Transsexual	16	16.75	7.53		
Problematic pornography consumption	Male	256	46.95	28.54	191.872	<0.001
	Female	404	25.27	11.42		
	Transsexual	16	75.25	37.00		
Satisfaction with sex life	Male	256	19.28	8.71	39.733	<0.001
	Female	404	22.23	9.38		
	Transsexual	16	9.50	2.37		

Regarding age, it was found that social media addiction was significantly higher among participants aged 18-25 (M = 15.54, SD = 5.66), followed by those aged 36-40 (M = 15.49, SD = 5.67) and 26-30 (M = 14.91, SD = 5.10), while the lowest levels were observed among those aged 41-45 (M = 12.33, SD = 4.20) (p = 0.022). Online sexual activity differed significantly across age groups, with the highest levels reported by individuals aged 31-35 (M = 7.78, SD = 4.86), 41-45 (M = 7.17, SD = 3.20), and 36-40 (M = 7.59, SD = 6.44). Lower levels were observed among participants aged 18-25 (M = 5.37, SD = 4.45) and those aged 50+ (M = 6.45, SD = 6.73) (p < 0.001). Problematic pornography consumption was also significantly higher among participants aged 41-45 (M = 38.52, SD = 9.97), followed by those aged 31-35 (M = 36.17, SD = 20.82) and 26-30 (M = 36.07, SD = 24.03). The lowest levels were found among participants aged 50+ (M = 25.73, SD = 26.56) (p = 0.001). Finally, satisfaction with sex life did not differ significantly by age group (p = 0.087) (Table 4).

**Table 4:** Age differences in social media addiction, online sexual activity, problematic pornography use, and satisfaction with sex life N: 676, n: frequency, M: mean, SD: standard deviation, H: H-statistic (df = 6), *: Kruskal-Wallis

	Age	n	M	SD	H (6)	p*
Social media addiction	18-25	196	15.54	5.66	14.833	0.022
	26-30	92	14.91	5.10		
	31-35	108	13.93	5.08		
	36-40	108	15.49	5.67		
	41-45	24	12.33	4.20		
	46-50	60	14.20	5.22		
	50+	88	13.98	4.95		
Online sexual activity	18-25	196	5.37	4.45	28.902	<0.001
	26-30	92	7.13	3.95		
	31-35	108	7.78	4.86		
	36-40	108	7.59	6.44		
	41-45	24	7.17	3.20		
	46-50	60	6.87	6.54		
	50+	88	6.45	6.73		
Problematic pornography consumption	18-25	196	33.00	18.88	23.600	0.001
	26-30	92	36.07	24.03		
	31-35	108	36.17	20.82		
	36-40	108	33.13	29.69		
	41-45	24	38.52	9.97		
	46-50	60	32.73	26.56		
	50+	88	32.59	28.67		
Satisfaction with sex life	18-25	196	20.31	9.66	11.058	0.087
	26-30	92	22.04	10.08		
	31-35	108	22.00	8.21		
	36-40	108	18.48	10.10		
	41-45	24	22.83	7.06		
	46-50	60	21.27	8.88		
	50+	88	21.18	8.24		

Regarding educational level, social media addiction was significantly higher among high school graduates (M = 15.68, SD = 5.41) than among university graduates (M = 14.20, SD = 5.60) and master’s degree holders (M = 14.33, SD = 4.72) (p = 0.003). Online sexual activity did not differ significantly across educational levels (p = 0.136). Problematic pornography consumption was significantly higher among high school graduates (M = 42.00, SD = 30.45), followed by university graduates (M = 31.50, SD = 19.47), and lowest among master’s degree holders (M = 29.48, SD = 15.65) (p = 0.004). Finally, satisfaction with sex life differed significantly across educational groups (p < 0.001), with master’s degree holders reporting the highest levels of satisfaction (M = 23.40, SD = 8.03), followed by university graduates (M = 20.34, SD = 9.10) and high school graduates (M = 19.51, SD = 10.02) (Table 5).

**Table 5:** Educational level differences in social media addiction, online sexual activity, problematic pornography use, and satisfaction with sex life N: 676, n: frequency, M: mean, SD: standard deviation, H: H-statistic (df = 2), *Kruskal-Wallis

	Educational level	n	M	SD	H (2)	p*
Social media addiction	High school graduate	236	15.68	5.41	11.457	0.003
	University graduate	272	14.20	5.60		
	Master's degree holder	168	14.33	4.72		
Online sexual activity	High school graduate	236	7.61	6.56	3.995	0.136
	University graduate	272	6.47	4.74		
	Master's degree holder	168	5.74	4.21		
Problematic pornography consumption	High school graduate	236	42.00	30.45	11.120	0.004
	University graduate	272	31.50	19.47		
	Master's degree holder	168	29.48	15.65		
Satisfaction with sex life	High school graduate	236	19.51	10.02	16.765	<0.001
	University graduate	272	20.34	9.10		
	Master's degree holder	168	23.40	8.03		

Regarding sexual orientation, social media addiction was highest among pansexual participants (M = 19.13, SD = 4.08), followed by bisexual individuals (M = 18.40, SD = 3.66), homosexual participants (M = 14.67, SD = 7.26), and heterosexuals (M = 14.26, SD = 5.24) (p < 0.001). Online sexual activity also differed significantly across groups (p < 0.001), with homosexual participants reporting the highest levels (M = 12.00, SD = 3.64), followed by pansexual (M = 11.25, SD = 6.28) and bisexual participants (M = 10.10, SD = 6.48). Heterosexuals reported the lowest levels (M = 5.98, SD = 4.96). Problematic pornography consumption was notably higher among bisexual participants (M = 47.30, SD = 30.95), followed by pansexual (M = 41.63, SD = 26.23) and homosexual participants (M = 37.17, SD = 25.98). The lowest levels were reported by heterosexuals (M = 33.30, SD = 22.69) (p < 0.001). Satisfaction with sex life also differed significantly (p < 0.001): homosexual participants reported the highest satisfaction (M = 27.50, SD = 6.12), followed by heterosexuals (M = 21.17, SD = 9.26), while pansexual (M = 16.63, SD = 10.45) and bisexual participants (M = 15.00, SD = 6.06) reported the lowest levels (Table 6).

**Table 6:** Sexual orientation differences in social media addiction, online sexual activity, problematic pornography use and satisfaction with sex life N: 676, n: frequency, M: mean, SD: standard deviation, H: H-statistic (df = 3), *: Kruskal-Wallis

	Sexual orientation	n	M	SD	H (3)	p*
Social media addiction	Heterosexual	580	14.26	5.24	51.936	<0.001
	Homosexual	24	14.67	7.26		
	Bisexual	40	18.40	3.66		
	Pansexual	32	19.13	4.08		
Online sexual activity	Heterosexual	580	5.98	4.96	69.605	<0.001
	Homosexual	24	12.00	3.64		
	Bisexual	40	10.10	6.48		
	Pansexual	32	11.25	6.28		
Problematic pornography consumption	Heterosexual	580	33.30	22.69	22.312	<0.001
	Homosexual	24	37.17	25.98		
	Bisexual	40	47.30	30.95		
	Pansexual	32	41.63	26.23		
Satisfaction with sex life	Heterosexual	580	21.17	9.26	34.980	<0.001
	Homosexual	24	27.50	6.12		
	Bisexual	40	15.00	6.06		
	Pansexual	32	16.63	10.45		

Correlations between social media addiction, online sexual activity, problematic pornography use, and satisfaction with sex life

Significant correlations were found between the main variables of the study. Social media addiction was positively correlated with online sexual activity (rs = 0.263, p < 0.001) and problematic pornography consumption (rs = 0.256, p < 0.001), while it was negatively correlated with satisfaction with sex life (rs = -0.230, p < 0.001). Online sexual activity was positively correlated with problematic pornography consumption (rs = 0.783, p < 0.001) and negatively correlated with satisfaction with sex life (rs = -0.148, p < 0.001). Problematic pornography consumption was also negatively correlated with satisfaction with sex life (rs = -0.293, p < 0.001) (Table 7).

**Table 7:** Correlations between social media addiction, online sexual activity, problematic pornography use, and satisfaction with sex life Spearman’s rank-order correlations, * p < 0.001

	Social media addiction	Online sexual activity	Problematic pornography consumption	Satisfaction with sex life	
Social media addiction	1.000				
Online sexual activity	0.263*	1.000			
Problematic pornography consumption	0.256*	0.783*	1.000		
Satisfaction with sex life	-0.230*	-0.148*	-0.293*	1.000	

Effects of social media addiction, online sexual activity, problematic pornography use, and satisfaction with sex life

A series of simple linear regressions was conducted to examine the predictive relationships between social media addiction, online sexual activity, problematic pornography consumption, and satisfaction with sex life. Social media addiction was a significant positive predictor of online sexual activity, F (1, 674) = 98.043, p < 0.001, accounting for approximately 13% of the variance (R² = 0.127). The regression coefficient indicated that higher social media addiction scores predicted greater online sexual activity (β = 0.356, p < 0.001). Similarly, social media addiction significantly predicted problematic pornography consumption, F (1, 674) = 133.447, p < 0.001, explaining 16.5% of the variance (R² = 0.165). Higher levels of social media addiction were associated with increased problematic pornography use (β = 0.407, p < 0.001). In contrast, social media addiction was a significant negative predictor of satisfaction with sex life, F (1, 674) = 42.797, p < 0.001, accounting for 6% of the variance (R² = 0.060). Greater social media addiction was associated with lower satisfaction with sex life (β = -0.244, p < 0.001) (Table 8).

**Table 8:** Results of simple linear regression analysis with social media addiction predicting online sexual activity, problematic pornography use, and satisfaction with sex life R²: coefficient of determination, F: F-statistic, B: unstandardized regression coefficient, β: standardized regression coefficient

Outcome variables	Social media addiction (predictor)				
	R^2^	F (1, 674)	B	β	p
Online sexual activity	0.127	98.043	0.357	0.356	<0.001
Problematic pornography consumption	0.165	133.447	1.802	0.407	<0.001
Satisfaction with sex life	0.060	42.797	-0.423	-0.244	<0.001

Online sexual activity was also a significant positive predictor of problematic pornography consumption, F (1, 674) = 854.900, p < 0.001, explaining 55.9% of the variance (R² = 0.559). Higher online sexual activity strongly predicted higher problematic pornography consumption (β = 0.748, p < 0.001). Furthermore, online sexual activity negatively predicted satisfaction with sex life, F (1, 674) = 32.705, p < 0.001, with 4.6% of the variance explained (R² = 0.046). Higher engagement in online sexual activity was associated with lower sexual satisfaction (β = -0.215, p < 0.001) (Table 9).

**Table 9:** Results of simple linear regression analysis with online sexual activity predicting problematic pornography use and satisfaction with sex life R²: coefficient of determination, F: F-statistic, B: unstandardized regression coefficient, β: standardized regression coefficient

Outcome variables	Online sexual activity (predictor)				
	R^2^	F (1, 674)	B	β	p
Problematic pornography consumption	0.559	854.900	3.306	0.748	<0.001
Satisfaction with sex life	0.046	32.705	-0.372	-0.215	<0.001

Problematic pornography consumption also significantly predicted lower satisfaction with sex life, F (1, 674) = 106.536, p < 0.001, accounting for 13.6% of the variance (R² = 0.136). Increased problematic pornography use was associated with reduced sexual satisfaction (β = -0.369, p < 0.001) (Table 10).

**Table 10:** Results of simple linear regression analysis with problematic pornography use predicting satisfaction with sex life R²: coefficient of determination, F: F-statistic, B: unstandardized regression coefficient, β: standardized regression coefficient

Outcome variable	Problematic pornography consumption (predictor)				
	R^2^	F (1, 674)	B	β	p
Satisfaction with sex life	0.136	106.536	-0.144	-0.369	<0.001

**Figure 1:**
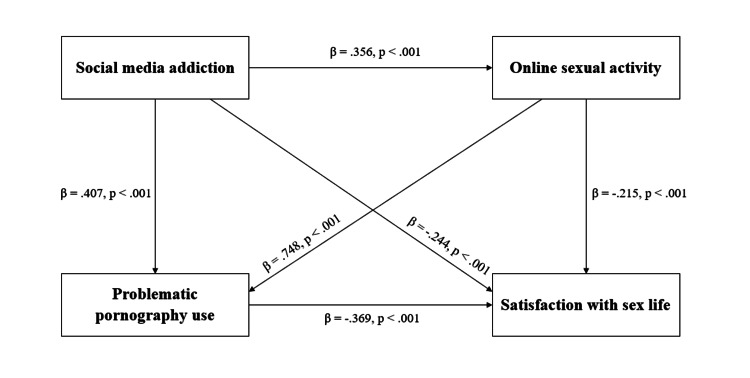
Effects between social media addiction, online sexual activity, problematic pornography use, and satisfaction with sex life Standardized regression coefficients (β) from simple linear regression analyses examining the effects of social media addiction, online sexual activity, problematic pornography use, and satisfaction with sex life. All paths shown are statistically significant at p < 0.001.

Figure 1 schematically shows the effects of social media addiction, online sexual activity, problematic pornography use, and satisfaction with sex life.

## Discussion

The present study explored the associations between social media addiction, online sexual activity, problematic pornography consumption, and satisfaction with sex life among Greek adults. The findings indicate a consistent pattern in which higher engagement in digital sexual behaviors and excessive media use correlate with lower sexual satisfaction, echoing a growing body of research addressing the consequences of digital sexual practices [10,33,34,37,39].

The positive relationship between social media addiction and online sexual activity aligns with findings by Nolin et al. [48], who noted that social media platforms often serve as gateways for sexual content and interactions, particularly among younger populations. Similarly, Koletić et al. [57] identified social networking platforms as significant predictors of online sexual behaviors, especially among individuals seeking instant gratification and emotional regulation. This suggests that digital environments may reinforce patterns of compulsive sexual exploration, potentially displacing in-person intimacy.

Moreover, social media addiction significantly predicted problematic pornography consumption. This relationship mirrors the conclusions drawn by Donevan et al. [58], who argued that excessive online engagement may foster dissociative and compulsive sexual behavior. Gryc et al. [59] similarly observed that digital hyperconnectivity is often accompanied by increased pornography use, particularly when social isolation or emotional dysregulation is present.

Consistent with previous studies [60,61], the results of the present study demonstrate that problematic pornography consumption is negatively associated with sexual satisfaction. Štulhofer et al. [62] emphasized that such use may lead to unrealistic sexual expectations, decreased emotional intimacy, and increased dissatisfaction with partnered sex. This aligns with the Acquisition, Activation, Application Model [63], which suggests that individuals may internalize pornographic scripts that become incompatible with real-life sexual experiences, leading to frustration or dissatisfaction.

Online sexual activity, although often perceived as a safe or exploratory outlet, was also associated with lower sexual satisfaction. These findings are in line with those of Ševčíková et al. [64], who found that even among older populations, pornography use was linked to diminished relationship satisfaction, particularly when it substituted for partnered sex. Similarly, Coyne et al. [65] reported that increased engagement with online sexual content can foster body image concerns and relational insecurities, which may indirectly diminish sexual satisfaction.

Interestingly, the adverse effect of social media addiction on sexual satisfaction has also been highlighted by Mari et al. [66], who demonstrated that compulsive use of social media disrupts emotional bonding and reduces the quality of romantic and sexual relationships. Varchetta et al. [67] added that digital addiction may blur boundaries between public and private intimacy, causing relational strain and dissatisfaction.

The strong predictive relationship between online sexual activity and problematic pornography consumption observed in this study is supported by findings from Miguel et al. [68], who noted a reinforcing loop between online sexual seeking and escalating pornography use. Yu et al. [69] argued that such escalation may reflect a broader addictive pattern wherein tolerance and compulsive use increase over time, accompanied by psychological distress and reduced satisfaction with offline sexual encounters.

In terms of demographic patterns, our findings showed age and gender disparities in digital sexual behaviors and satisfaction levels. These patterns are consistent with the literature [42,70], indicating that younger individuals are more likely to engage in online sexual activity and exhibit higher levels of social media and pornography use. Educational level also emerged as a meaningful factor in participants’ digital sexual behaviors and satisfaction. In line with findings by Bayat et al. [71] and Tian et al. [72], participants with lower educational attainment, specifically high school graduates, reported significantly higher levels of social media addiction, problematic pornography consumption, and online sexual compulsivity. Conversely, those with a master’s degree consistently reported the highest satisfaction with sex life and the lowest levels of problematic use, supporting prior evidence [73] that education may act as a protective factor through increased digital literacy, emotional regulation, and sexual self-awareness. At the same time, females and transgender individuals were disproportionately affected by lower sexual satisfaction, potentially due to the compounded effects of digital objectification and minority stress [74,75].

Sexual orientation also emerged as a relevant factor, with sexual minorities reporting lower levels of sexual satisfaction compared to heterosexual participants. Specifically, homosexual participants reported higher levels of sexual satisfaction compared to bisexual and pansexual participants, who showed the lowest satisfaction scores. This indicates that sexual minorities are not a homogeneous group, and their experiences with sexual well-being may differ substantially across subgroups. This is in accordance with previous work by Björkenstam et al. [76] and Komlenac and Hochleitner [77], who reported that sexual minorities may experience additional barriers to satisfaction, including internalized stigma, limited access to affirming sexual education, and greater exposure to digital sexual spaces that do not align with their relational goals. However, our results suggest that homosexual individuals may experience less of this disparity than bisexual or pansexual participants. One possible explanation is that homosexual participants may have greater community support and more frequent engagement in same-orientation partnerships, which may buffer against dissatisfaction. In contrast, bisexual and pansexual individuals may face additional challenges, such as identity invalidation or relational mismatch, that could intensify the negative impact of problematic digital sexual behaviors on satisfaction. These differences highlight the importance of examining sexual minority groups separately rather than aggregating them into a single category.

Conversely, some research suggests more nuanced or even positive interpretations of digital sexual engagement. For instance, Ševčíková et al. [64] and Komlenac and Hochleitner [77] noted that pornography use, when not problematic, may increase sexual flexibility and exploration, especially among women. However, these benefits are likely moderated by contextual factors such as consent, emotional readiness, and relationship dynamics.

Notably, our regression analyses showed that all three variables, social media addiction, online sexual activity, and problematic pornography consumption, were significant negative predictors of satisfaction with sex life. These findings contribute to an expanding field [78,79] that highlights how digitally mediated sexual practices, while normalized, may paradoxically reduce subjective fulfillment and relational intimacy.

Finally, the broader sociocultural context of Greece may also be important in understanding these dynamics. As suggested by Pawlikowska-Gorzelańczyk et al. [80], cultural conservatism around sexuality can create tension between private digital exploration and public sexual norms, thereby amplifying dissatisfaction or guilt.

Limitations and strengths

While the present study provides important insights into the interplay between digital behaviors and sexual satisfaction, several limitations should be acknowledged. First, the study did not assess the frequency of pornography consumption nor explore the specific content categories participants viewed. These dimensions may influence both the degree of problematic use and its psychological or relational effects, as specific genres or consumption patterns may have distinct outcomes. Similarly, although social media addiction was measured, the study did not account for which platforms participants used most frequently, a factor that could influence the nature and intensity of both social and sexual engagement online. Second, the sample was not demographically balanced, with female participants comprising the majority and heterosexual individuals representing an overwhelming proportion of respondents. This demographic skew may limit the generalizability of findings to more diverse populations, including sexual and gender minorities whose experiences with digital intimacy and satisfaction may differ significantly. Moreover, recruitment was conducted entirely through online platforms, which may introduce sampling bias by disproportionately attracting individuals who are more digitally active or more comfortable with online environments. Additionally, the study relied exclusively on self-report questionnaires, which may be subject to social desirability bias, recall limitations, or underreporting of sensitive behaviors. Finally, the cross-sectional design precludes causal interpretations, as the temporal direction of associations between digital behaviors and sexual satisfaction cannot be determined.

Despite these limitations, the study also possesses several notable strengths. To our knowledge, it is one of the few investigations to examine social media addiction, online sexual activity, problematic pornography consumption, and sexual satisfaction simultaneously within the same analytical framework, allowing for a more integrated understanding of digital sexual behaviors. The use of validated psychometric instruments with excellent reliability further strengthens the methodological rigor. Additionally, the large sample size enhances statistical power, and the inclusion of diverse demographic groups provides valuable insight into how digital sexual behaviors vary across age, education, and sexual orientation. Significantly, the study contributes culturally specific evidence from Greece, an underrepresented context in digital sexuality research, thereby enriching the international literature with findings from a Southern European population.

Practical implications and suggestions for further research

The findings of this study carry important practical implications for both sexual health professionals and digital behavior researchers. Given the observed associations between social media addiction, online sexual activity, problematic pornography consumption, and reduced sexual satisfaction, intervention programs should incorporate digital literacy and media regulation strategies into sexual health education. Clinicians and counselors working with individuals or couples experiencing sexual dissatisfaction may benefit from screening for excessive online sexual behaviors and problematic digital media use, which are often overlooked yet relevant contributors to relational distress. Moreover, public health campaigns and educational initiatives could focus on promoting balanced digital engagement and fostering awareness around the potential disconnect between online sexual experiences and real-life intimacy. These initiatives are particularly significant in Greece, where traditional family values, collectivistic social norms, and lingering stigma around sexual communication may influence how individuals engage with and interpret digital sexual content. As such, interventions should be culturally adapted, taking into account the tension between conservative norms and the rapid digitalization of intimacy in contemporary Greek society. Potential actionable interventions may include structured workshops on healthy digital boundaries, school-based curricula addressing online sexual behaviors and media influences, couple-focused programs that support open communication about digital habits, and community-level campaigns promoting safe and context-appropriate online sexual practices. Additionally, clinicians may integrate brief digital-use assessments into routine care and offer psychoeducation aimed at reshaping unrealistic expectations formed through online sexual exposure. This is particularly pertinent for younger users, who, as this and previous studies suggest, are more vulnerable to forming maladaptive sexual scripts through frequent exposure to digital sexual content.

Future research should address the limitations noted above by including measures of pornography frequency, content categories, and platform-specific social media use. Such granularity could offer deeper insights into the mechanisms that link digital behavior with sexual satisfaction. Additionally, qualitative or mixed-method approaches may be beneficial in capturing the nuanced emotional and relational experiences associated with digital intimacy practices. Moreover, future research should consider how cultural expectations, religious influences, and gender norms shape digital sexual behaviors in Greece, as these factors may moderate both engagement patterns and their association with sexual satisfaction. Another promising direction would be to investigate couples’ dynamics, such as how shared or divergent patterns of pornography use and social media engagement affect mutual satisfaction, trust, and communication. Finally, studies could also assess the impact of emerging technologies, such as AI-generated sexual content, virtual reality pornography, or gamified intimacy apps, on sexual satisfaction and relational functioning in both individuals and romantic dyads.

## Conclusions

This study offers important empirical insights into how social media addiction, online sexual activity, and problematic pornography consumption are associated with sexual satisfaction among Greek adults. The findings demonstrate consistent patterns in which higher engagement in digital sexual behaviors and compulsive online use predict lower levels of satisfaction with sex life. These associations were evident across multiple measures and demographic groups, suggesting that digitally mediated sexual engagement, while increasingly normalized, may carry unintended psychological and relational costs. By focusing on a Greek sample, this study contributes culturally specific evidence to a growing international body of literature examining the intersection of digital behavior and sexual well-being. As online environments continue to shape contemporary sexual norms, understanding the nuances of these relationships becomes essential for researchers, clinicians, and educators alike. The results underscore the need for further investigation into the mechanisms and contexts that shape digital intimacy, as well as the development of targeted interventions to promote healthy sexual functioning in a rapidly digitizing world.
